# Addressing inequities in the global burden of maternal undernutrition: the role of targeting

**DOI:** 10.1136/bmjgh-2019-002186

**Published:** 2020-03-18

**Authors:** Parul Christian, Emily R Smith, Anita Zaidi

**Affiliations:** 1 Johns Hopkins University Bloomberg School of Public Health, Baltimore, Maryland, USA; 2 George Washington University, Washington, DC, USA; 3 Harvard University T H Chan School of Public Health, Boston, Massachusetts, USA; 4 Bill and Melinda Gates Foundation, Seattle, Washington, USA

**Keywords:** nutrition, public health

**Summary box**

- Recent estimates of low birth weight (LBW) (weight <2500 g) indicate the burden to be high; 20.5 million babies are born too small annually, although data from low-income countries is sparse.
- The WHO’s antenatal care guidelines recommend supplementation with ‘balanced energy and protein’ (BEP) during pregnancy in undernourished settings (where >20% women are too thin based on their body mass index).
- Motivated by equity, we make the case for targeting individual, higher-risk women for BEP supplementation, which would be safe, affordable and likely more impactful by giving all vulnerable women access to this effective intervention.
- Innovative, programmatic action using such a precision public health approach is needed to improve women's health and accelerate progress towards the 2030 target for reducing LBW by 30%.

The burden of maternal undernutrition is well defined, but global momentum and political will to address it is lacking. Maternal nutrition has long been neglected largely due to gender-based inequities in resource allocation. The first 1000 days, especially the period from conception through birth (approximately the first 280 days), is a critical window for future growth, development and resilience in the face of possible adversity or disease. Despite this biological imperative, the bulk of resources allocated to maternal and child health have been directed towards the child^1^—with little attention to a woman’s nutritional status prior to or during pregnancy.

**Figure 1:**
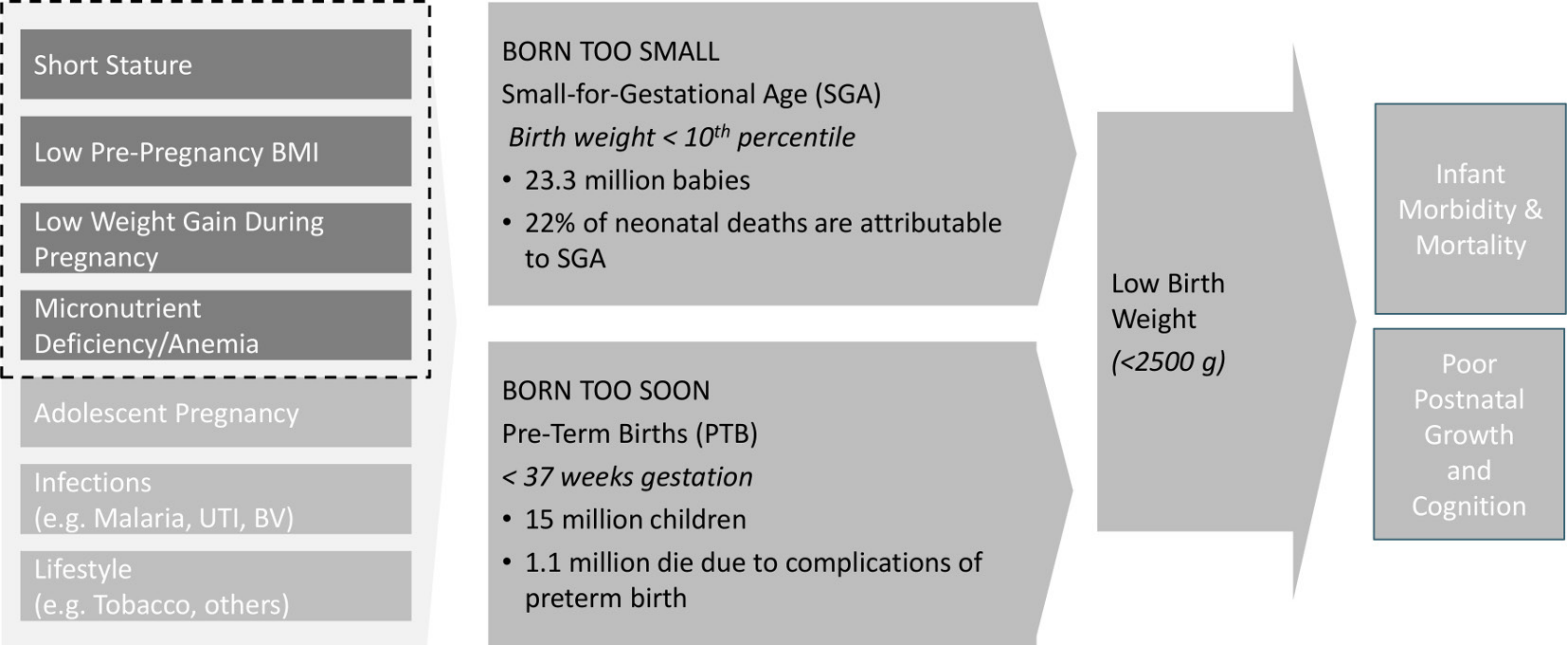
Maternal nutrition and other factors influencing adverse birth outcomes. BMI, body mass index; UTI, urinary tract infection; BV, bacterial vaginosis.

A recent estimate noted that 20.5 million babies are born ‘low birth weight’ (LBW <2500 g) every year; 48% of these are born in South Asia and 24% in sub-Saharan Africa.^2^ Babies born small at birth have an increased risk of mortality, morbidity and suboptimal growth and cognitive development throughout childhood, thus perpetuating the intergenerational cycle of growth failure. Progress towards reducing LBW was somewhat rapid from 2000 to 2009, but it stalled in the past decade to only 1% average annual risk reduction (AARR).^2^ The World Health Assembly target of 30% reduction in LBW by 2025 is unreachable at this rate of change; the AARR must nearly triple to 2.7% to meet the global goal. This high burden of LBW is biologically linked to the high burden of global maternal undernutrition; 450 million women in low-income and middle-income countries (LMIC) are estimated to have short stature,^3^ 240 million are underweight (body mass index (BMI) <18.5),^4^ and 468 million are anaemic.^5^ India faces the highest burden of maternal undernutrition both proportionately and in absolute terms; 100 million adult women in India have low BMI (<18.5).^4^ These maternal factors are causally linked with LBW and its two underlying causes—preterm birth and small-for-gestational age (SGA) (figure 1), although it must be noted that having quality data for these requires accurate gestational age assessment, which is relatively uncommon in LMIC.

The WHO recently updated the guidelines for antenatal care for a ‘healthy pregnancy experience’. These guidelines offer evidence-based recommendations to support nutrition in pregnancy.^6^ WHO’s recommendations include nutrition counselling for a healthy diet and adequate weight gain in pregnancy, daily use of iron–-folic acid supplement to prevent anaemia, and calcium supplementation to reduce the risk of pre-eclampsia in low calcium intake settings. Additionally, new evidence has demonstrated efficacy and superiority of prenatal multiple micronutrient supplementation versus iron–folic acid,^7 8^ calling for the need to revise current guidelines. Finally, based on a systematic review of evidence,^9^ the guidelines also include a ‘context-specific’ recommendation that pregnant women in ‘undernourished populations’ consume balanced energy and protein (BEP) supplements. BEP refers to ready-to-use or ready-to-be-cooked foods provided daily to supplement home-based diets to increase energy and protein intake in pregnancy. This recommendation recognises that, in many LMIC settings, women begin a pregnancy with low BMI. Women with chronically deficient diets are at higher risk for inadequate weight gain in pregnancy; this is a modifiable risk factor linked to poor fetal growth. Furthermore, nutritional requirements for protein, energy and many micronutrients increase during pregnancy, and they are often unmet without substantially increased dietary intake and improved dietary diversity. The BEP guideline is meant to address these nutritional gaps. The systematic review showed BEP to reduce SGA by 21%,^9^ and increase mean birth weight by 41 g; the impact of BEP on increasing birth weight was higher in undernourished women (approximately 100 g).^10^

**Figure 2:**
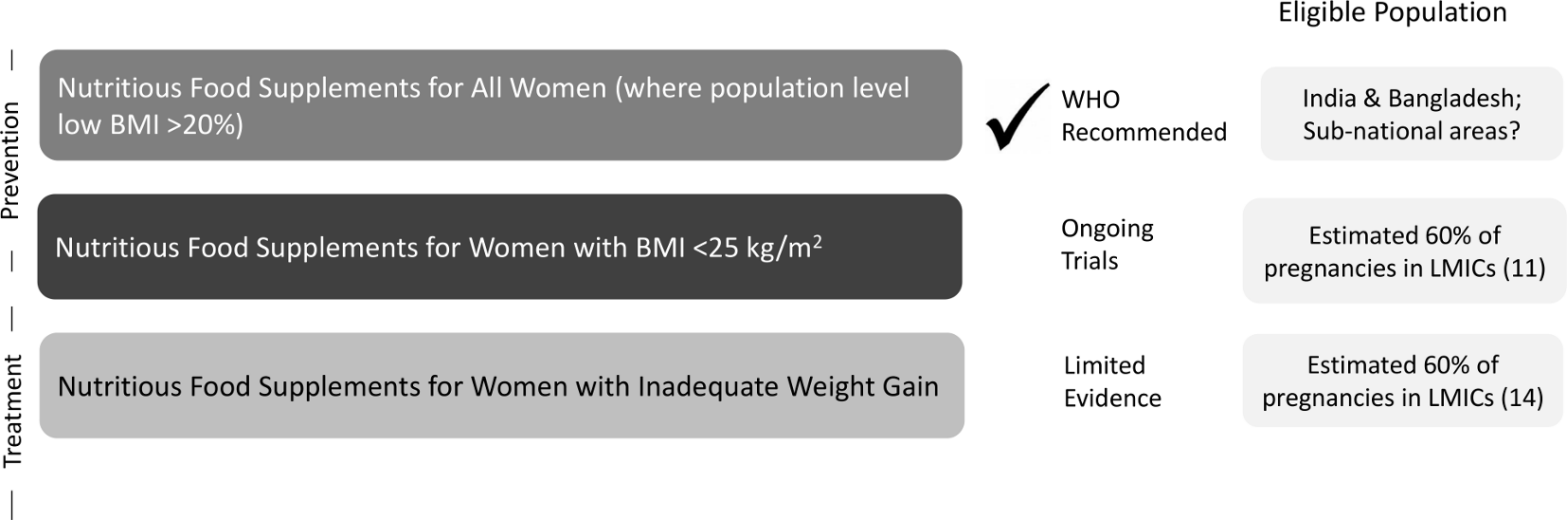
Recommended and alternative use cases for nutritious food supplements in pregnancy in undernourished contexts. BMI, body mass index; LMIC, low-income and middle-income countries.

The WHO guideline recommends a population-based approach and suggests use of BEP where the population prevalence of low BMI (<18.5) is greater than 20% (figure 2). This recommendation arises in part from concerns related to global trends of increasing BMI. Mean BMI in LMIC is rising.^11^ Except for Sub-Saharan Africa, this trend is as common in rural contexts as it is in cities.^12^ While the obesity epidemic is an increasingly important public health problem, there is not clear evidence that BEP during pregnancy increases the risk of obesity. Further, the current guideline applies nationally to only two countries—India and Bangladesh—where the prevalence of low maternal BMI is >20%.^4^ This means that women in undernourished or food insecure regions of other countries are not eligible for BEP; thus, the current guideline ignores the substantial sub-national heterogeneity in maternal nutritional status. We advise more countries should consider sub-national geographic targeting, but guidance on this is limited.

Surprisingly, the current WHO guideline advises against identification and supplementation of specific undernourished pregnant women. That recommendation likely arises from a focus on equality and perceived ease of implementation. However, we argue that a targeted, equity-focused approach is safe, may be more impactful and would potentially be more affordable than a population-approach.

Using a person-specific guideline would facilitate implementation, impact and equity through two additional use cases for BEP (figure 2). First, women could be targeted for BEP based on their pre-pregnancy or early pregnancy BMI. One approach could be to target only the most undernourished (<18.5 BMI), but arguably, this would not fully address the entire burden of poor fetal growth as inadequate weight gain can occur in women regardless of their baseline BMI. A meta-analysis that included pregnant women in high-income and middle-income countries, showed that inadequate gestational weight gain is associated with the highest risks of preterm birth among women with pre-pregnancy BMI <25.^13^ Thus, we argue that women with BMI <25 are likely to benefit from a food supplement in pregnancy in undernourished, food insecure contexts. Each country should decide the upper cut-off of BMI, depending on the burden of maternal undernutrition and related adverse pregnancy outcomes. A second targeting approach might be based on inadequate gestational weight gain during pregnancy, which is estimated to be high in LMICs.^14^ While this is a biologically sound approach to using BEP, there is currently no evidence about the efficacy, feasibility, or acceptability of this type of targeting strategy.

We posit three major motivations for adopting a maternal BMI targeted approach. First, it would avoid concerns related to the risk of providing food supplements to overweight and obese women. Second, evidence from efficacy trials categorised by high and low burden of undernutrition would suggest that undernourished women are most likely to benefit.^10^ Third, this approach would be more cost-effective and many more at-risk women would have access to this effective intervention than is achievable with the current guidelines. In a world that is increasingly heterogenous, we must question global ‘one-size-fits all’ guidelines^15^; precision public health approaches are urgently needed. Segmentation, targeting and applying an equity (not equality) lens is likely to yield high impact, cost-effective outcomes that will aid us in reaching the Sustainable Development Goal targets faster. The Bill & Melinda Gates Foundation is currently funding ongoing research in South Asia and Africa testing ready-to-use food supplements for pregnant (and lactating) women designed to meet the macro (protein and energy) and micronutrient specifications as set by an expert group^16^ and targeting <25 BMI women; these trials will generate further evidence on the impact on birth outcomes (figure 2).

An increasing recognition of the intergenerational nature of health and development may lead some to suggest that it is insufficient to simply improve nutrition in pregnancy, arguing that improving health during preconception, adolescence, early childhood, or even when the mother herself was a fetus is necessary to fully optimise health in the future offspring. However, as argued by Garza,^17^ it is unlikely that growth constraints in parents in utero or as children could explain a high proportion of current child growth failure and that increases in growth can be achieved within a generation with appropriate nutrition and care despite the adversities faced by the previous generation.^18^ While social and economic development, as well as gender equity, are essential for women and children to achieve their full potential, it is not a reason to deprioritise an effective, ready-to-implement intervention that can begin to address the intergenerational nature of growth failure. Applying an equity and gender lens to the problem, many millions of women who become pregnant each year should not be deprived the right and chance to have healthier babies.

Both as individuals and as influencers of the health and well-being of their families, women and girls hold roles in their community that make them critical drivers of development. Receiving the appropriate nutrition is essential for them to reach their potential and meaningfully contribute to their communities—by succeeding in school, achieving their maximum work productivity, and having their own healthy children, if they so choose. We advocate for targeted balanced energy and protein supplementation for pregnant (and lactating) women in low-income and food insecure contexts as an important strategy, alongside use of micronutrient supplementation and nutrition counselling, for changing the health trajectories of current and future generations.
